# Significance of Intermittent Hypoxic Episodes in Premature Infants Prior to Discharge

**DOI:** 10.7759/cureus.36113

**Published:** 2023-03-14

**Authors:** Saleh Alalaiyan, Deena Shakeeb, Fahad Al Hazzani, Abdulaziz Binmanee

**Affiliations:** 1 Pediatrics, King Faisal Specialist Hospital and Research Centre, Riyadh, SAU; 2 Pediatrics/Neonatology, King Faisal Specialist Hospital and Research Centre, Riyadh, SAU

**Keywords:** neonatal outcome, caffeine therapy, intermittent hypoxia, apnea of prematurity, neonates

## Abstract

Objective

The aim of this study was to determine the rate and severity of intermittent hypoxic episodes in premature infants who underwent overnight pulse oximetry prior to discharge.

Methods

Preterm infants with a birth weight of 1500 grams or less and who underwent overnight pulse oximetry prior to discharge were included. Maternal and neonatal demographic data and complications of prematurity were recorded. All infants underwent overnight pulse oximetry prior to discharge and the McGill score was used to categorize the degree of desaturations (categories 1-4; normal, mildly, moderately, and severely abnormal).

Results

Fifty infants underwent the overnight pulse oximetry The McGill score showed that 2% had no hypoxia, 50% had mild hypoxia, 20% had moderate hypoxia, and 28% had severe hypoxia. The frequency of desaturations (62.5%) was found more in infants with a birth weight of 1000 grams or less. The results showed that the O2 requirement at discharge was significant (p = 0.0341), and increased values of O2 at discharge were associated with more severe hypoxia. As a result of these findings, 40% of infants were discharged home on oxygen and 26% were discharged on caffeine. Fifty-two percent of infants were initially diagnosed to have stages 1 & 2 retinopathy of prematurity (ROP), 14% had stage 3, and 2% had stage 4 ROP. Eight percent of infants required surgical intervention for ROP.

Conclusions

Clinically inapparent significant episodes of intermittent hypoxia (IH) are frequent in preterm infants in the early postnatal age, and they may persist post-discharge. Knowledge of the association between IH and morbidity among all neonatal intensive care unit (NICU) caregivers would be of great benefit. Indications for screening preterm infants at risk of severe IH should be reconsidered.

## Introduction

Intermittent hypoxic (IH) episodes are due to immature respiratory control and are triggered by the cessation of respiratory neural output. Furthermore, their significance is commonly undervalued by clinically used pulse oximeters. A considerable problem has been reported regarding the inability to determine the incidence and magnitude of apneic events utilizing the standard pulse oximetry technique because it cannot document the obstructed inspiratory efforts that commonly prolong central respiratory pauses [1]. In extremely low birth weight infants, the incidence of IH progressively increases over the first weeks of postnatal life and declined over time. Moreover, increased oxygen-sensitive peripheral chemoreceptor activity has been associated with a higher incidence of apnea of prematurity [2].

These intermittent hypoxic episodes can be unapparent and easily missed by neonatal intensive care unit (NICU) caregivers, particularly prior to discharge. Hunt et al. showed in longitudinal home recordings of oxygen saturation utilizing pulse oximetry during unperturbed sleep in preterm and term infants that clinically unapparent IH occurs in epochs unperturbed by and temporally unrelated to apnea or bradycardia events, especially in preterm infants at 36 to 44 weeks postmenstrual age (PMA) [3]. They recommend performing more studies in preterm infants to better understand the frequency and severity of IH before and after NICU discharge, the neurodevelopmental outcome of these infants, and the clinical approach to ameliorating these episodes. Hypoxic episodes secondary to persistent apnea and bradycardia have been associated with disorders in their longer-term neurodevelopment [4]. Underdevelopment of the lung and the resultant lung injury that occurs in premature infants concurrent with respiratory instability can be responsible for frequent episodes of profound and recurrent hypoxemia [5].

In animal models, intermittent hypoxia occurring during a period of critical brain development evokes behavioral and neurochemical alterations that are long-lasting and consistent with disorders of minimal brain dysfunction [6].

The irregular respiratory pattern of premature infants becomes more regular as gestational age increases. The persistent apnea of extremely low birth weight infants contributes to their prolonged hospitalization. Some premature Infants continued to exhibit apnea and intermittent hypoxia beyond 37 weeks postmenstrual age. A prospective randomized clinical trial showed that substantial IH persists after the termination of caffeine treatment and progressively decreases with increasing PMA. Moreover, extended caffeine treatment decreases IH in premature [7] and late preterm infants [8].

In this descriptive study, we aimed to determine the rate and severity of intermittent hypoxic episodes in premature infants who underwent overnight pulse oximetry prior to discharge.

## Materials and methods

This is a retrospective descriptive study that included preterm infants with a birth weight of 1500 grams or less, and who had overnight pulse oximetry prior to discharge between January 2014 and December 2017. Infants with major congenital anomalies were excluded from the study. Maternal medical records were reviewed to obtain the following data: maternal age, antenatal care, antenatal steroids, use of magnesium sulfate, evidence of chorioamnionitis, evidence of hypertension during pregnancy, mode of delivery, and multiple gestations. Neonatal medical records were reviewed to obtain the following data: demographic data (gestational age, birth weight, sex), initial steps of resuscitation performed on infants, types of respiratory support used during the early hospital course, neonatal medications used for various indications (surfactant, caffeine citrate, steroids for bronchopulmonary dysplasia (BPD), and indomethacin), sepsis, length of hospital stay and complications of prematurity that included: respiratory distress syndrome (RDS), BPD at 36 weeks' postmenstrual age, patent ductus arteriosus (PDA), necrotizing enterocolitis (NEC), retinopathy of prematurity (ROP), intraventricular hemorrhage (IVH) and periventricular leukomalacia (PVL). In our NICU, all infants with a birth weight of 1500 grams or less should be started on a loading dose of 20 mg/kg caffeine citrate, followed by 5-8 mg/kg/day once daily. Caffeine therapy is usually stopped when infants reach 35 weeks corrected age and are discharged home when they are apnea-free for five days [9].

The pulse oximeter used in this study was a Criticare 504DX, from Criticare Systems Inc., Waukesha, Wisconsin. The recorded O2 saturation and heart rate data were stored in the oximeter and subsequently connected to the serial port of a PC. The application installed in the computer is able to transfer, store, analyze, and quantify the oximeter data.

The results of the pulse oximetry study were reviewed by a pediatric pulmonologist to confirm its significance. The McGill score was used to categorize the degree of desaturations (categories 1-4; normal, mildly, moderately, and severely abnormal) [10-12]. The score objectifies consensus opinion as to what constitutes a mildly, moderately, or severely abnormal oximetry result. The score correlates with the severity of hypoxic episodes [10]. The McGill score classifies hypoxia into three drops: <90%, <85%, and <80%. Furthermore, the definitions of mild, moderate, and severe as per the scoring system are as follows: mild hypoxia when infants have ≥3 drops in <90%, ≤3 drops in <85% but zero drops in <80%, moderate hypoxia when infants have ≥3 drops in <90%, >3 drops in <85%, and ≤ drops in <80%, and severe hypoxia when infants have ≥3 drops in <90%, >3 drops in <85% and >3 drops in <80%.

The definitions used in this study for BPD [13], NEC [14], IVH [15], ROP [16], and PVL [17] are in the appendix.

Statistical methods and analysis

Descriptive analyses were conducted on all obtained variables, utilizing Stata version 17.0 (StataCorp LLC, College Station, TX). These analyses involved the calculation of measures of centrality and variation for continuously scaled variables and the calculation of counts and percentages for categorically scaled variables. Graphically, box-and-whisker and stem-and-leaf plots illustrated the distributions of each of the variables. Where possible (i.e., given the nature of the data), pairwise Spearman correlation coefficients were calculated between all pairs of variables.

## Results

Fifty premature infants who had overnight pulse oximetry were identified and enrolled in the analysis. All infants had overnight pulse oximetry because they experienced short episodes of desaturation during feeding or sleeping except six infants with a median birth weight of 633.5 grams (540-1060) who had the study merely because they were born less than 27 weeks gestation.

**Figure 1:**
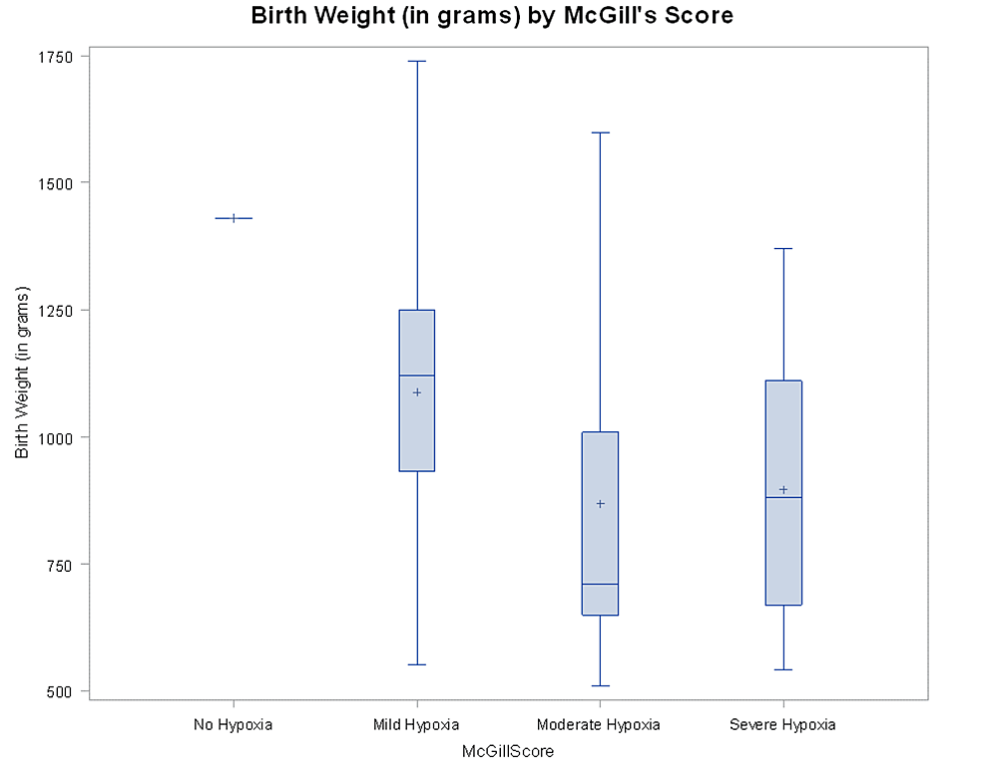
Categories of hypoxia severity determined by the McGill oximetry score

**Figure 2:**
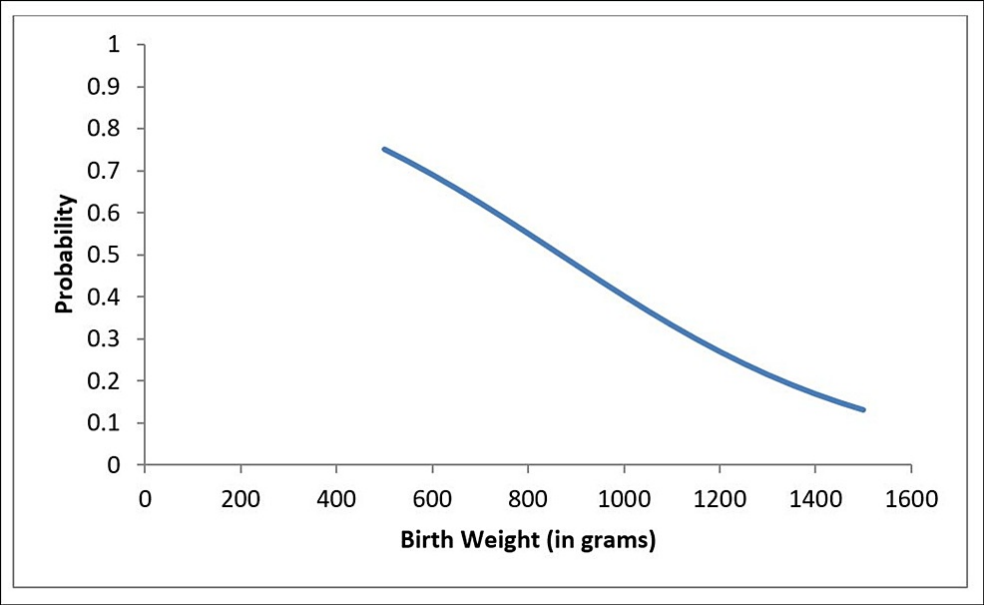
The relationship between hypoxic episodes and the birth weight

The maternal characteristics showed that maternal age (Mean ± SD) was 30.3 ± 5.34, 98% of mothers had prenatal care, 82% received antenatal steroids, 30% had antenatal magnesium sulfate, 12% had hypertension, 12% had evidence of chorioamnionitis, and 35% had a cesarean section to delivery these infants. Clinical characteristics of infants (Table 1), showed that gestational age (Mean ± SD) was 28.36 ± 2.59 weeks, birth weight (Mean ± SD) was 996.8±305.7 grams, and 52% were males. Eighty-four percent of infants required surfactant therapy for RDS, 60% were intubated in the delivery room, 80% were ventilated with conventional ventilation, 28% required high-frequency oscillatory ventilation (HFOV), 90% were started on caffeine, and 26% were discharged on caffeine. The McGill score showed that 2% had no hypoxia, 50% had mild hypoxia, 20% had moderate hypoxia, and 28% had severe hypoxia. The McGill score was dichotomized as Moderate/Severe vs No/Mild, and another set of logistic regression analyses was carried out. Additionally, it was found that O2 at discharge was significantly related to more hypoxia (moderate/severe) (P = 0.0028) (Table 2). The frequency of desaturations (62.5%) was found more in infants with a birth weight of 1000 grams or less. Furthermore, it was also found that decreased birth weight was significantly associated with moderate/severe hypoxia (p = 0.0157) (Figures 1-2). Thirty-three (66%) infants were diagnosed to have chronic lung disease and were weaned gradually off oxygen as preparation for discharge. 

**Table 1:** Clinical characteristics of infants GA: gestational age, BW: birth weight, nCPAP: nasal continuous positive pressure, HFNC: high-flow nasal cannula, HFOV: high-frequency oscillatory ventilation, iNO: inhaled nitric oxide, CLD: chronic lung disease, PDA: patent ductus arteriosus, NEC: necrotizing enterocolitis, ROP: retinopathy of prematurity, IVH: intraventricular hemorrhage, PVL: periventricular leukomalacia

GA (Mean ± SD)		28.36 ± 2.59
BW (Mean ± SD)		996.8±305.7
Males, n (%)		26 (52%)
Inborn, n (%)		48 (96%)
Intubation at birth, n (%)		30 (60%)
Epinephrine, n (%)		3 (6%)
Cardiac compression, n (%)		4 (8%)
nCPAP, n (%)		16 (32%)
Conventional ventilation, n (%)		40 (80%)
HFNC, n (%)		7 (14%)
HFOV, n (%)		14 (28%)
Surfactant, n (%)		42 (84%)
iNO, n (%)		3 (6%)
Caffeine, n (%)		45 (90%)
Discharged home on caffeine, n (%)		13 (26% )
O2 at 36 weeks, n (%)		33 (66%)
Steroids For CLD, n (%)		5 (10%)
Discharged on O2, n (%)		20 (40%)
RDS, n (%)		46 (92%)
Pneumothorax, n (%)		2 (4%)
PDA	Total, n (%)	17 (34%)
	Treated with ibuprofen, n (%)	9 (18%)
	Surgically ligated, n (%)	6 (12%)
NEC, n (%)		9 (18%)
Sepsis or sepsis-like syndrome, n (%)		32 (64%)
ROP	stage 1 & 2, n (%)	26 (52%)
	Stage 3, n (%)	7 (14%)
	Stage 4, n (%)	1 (2%)
	Required surgery, n (%)	4 (8%)
Seizure, n (%)		4 (8%)
IVH	Grade 1 & 2, n (%)	9 (18%)
	Grade 4, n (%)	5 (10%)
PVL, n (%)		8 (16%)
Hospital stay (days) median (range)		90 (28-193)

**Table 2:** Logistic regression analyses of the McGill score: Moderate/Severe vs No/Mild in relation to O2 at discharge

	P value
No hypoxia, n (%)	1 (2)	
Mild hypoxia, n (%)	25 (50)	
Moderate hypoxia, n (%)	10(20)	0.0028
Severe hypoxia, n (%)	14 (28)	
Oxygen at discharge, n (%)	20 (40)	

Fifty-two percent of infants were initially diagnosed to have stages 1 and 2 ROP, 14% had stage 3, and 2% had stage 4 ROP. Eight percent of infants required surgical intervention for ROP. Sixty-four percent of infants were started on antibiotics for sepsis or sepsis-like syndrome. Twenty-eight percent of infants had IVH, of these 10% had grade 4 and 16% had PVL. The average hospital stay among all infants was 90 (28-193) days.

## Discussion

The results of this study show several findings regarding IH in infants born prematurely. Brief episodes of oxygen desaturation may appear clinically insignificant to the NICU caregivers; however, in this study, we found that 48% of the studied infants had moderate and severe hypoxia. Twenty-six percent (26%) of the studied infants were discharged home on caffeine. We also found that decreased birth weight was significantly associated with moderate/severe hypoxia (p = 0.0157). These hypoxic episodes are usually due to immature respiratory control that results in apnea and respiratory pauses. Nevertheless, these episodes of IH were typically not apparent clinically and continuous physiologic recording was required to detect and confirm their significance. Intermittent hypoxia should be taken seriously because it might have adverse effects on neonatal outcomes. Studies show continuing exposure to IH during early postnatal life disrupts expression patterns of proteins involved with dopamine signaling [18] and causes a proinflammatory response, including increased levels of tumor necrosis factor-alpha and interleukin-1b [19]. These changes could result in multiple poor outcomes, including ROP and neurodevelopmental impairment. In this study, 16% of infants had stages 3 and 4 ROP and 8% of them required surgical intervention. In a previous report from our NICU, the rate of severe ROP was 8%, which is much less than the rate of ROP among our population in this study [20]. Although it is known that early exposure to high oxygen is a major risk factor for ROP, there is adequate evidence that IH can induce more production of vascular endothelial growth factor (VEGF) [21]. VEGF is a major mediator of the pathological angiogenesis observed in ROP [22].

The effect of IH on the neurodevelopment of these infants has been studied. In a multicenter Canadian Oxygen Trial (COT) using continuous recordings of oxygen saturation in a large cohort study, authors found a significant correlation between prolonged hypoxemic episodes during the first two to three months after birth and adverse 18-month outcomes, including late death or disability, cognitive language delay, and motor impairment [23]. Their results showed that the odds of developing cognitive or language impairment at 18 months corrected age was three times higher in infants who spent more time with events where pulse oximeter saturation (SpO2) was <80% for ≥1 min during their first 10 postnatal weeks compared to those who had very few such events after birth.

We discharged 26% of infants home on caffeine because they had significant hypoxic episodes during the overnight pulse oximetry study. Caffeine intake in preterm infants may have a neuroprotective effect by blocking adenosine action, increasing myelination, promoting oligodendroglial maturation, and process arborization in hypoxia-exposed white matter [24]. A prospective randomized clinical study that showed extending caffeine treatment to 40 weeks postmenstrual age decreased IH in premature infants [7]. There are still issues concerning optimal caffeine doses for infants. In a study aimed to determine the most effective dose of caffeine citrate for the prevention of IH in late preterm infants, authors found that 20 mg/kg/day of caffeine was the most effective dose in reducing IH in late preterm infants at two weeks of age [8].

In conclusion, clinically inapparent significant episodes of IH are frequent in preterm infants in early postnatal ages and they may persist to post-discharge. Knowledge of the association between IH and morbidity among all NICU caregivers would be of great benefit. Screening high-risk premature infants with continuous recordings of oxygen prior to discharge will help identify those who have severe episodes of IH to determine the appropriate treatment to be used.

The limitations of this study are the small sample size, being single-armed, and the lack of a control group. There was no long-term follow-up to assess neurodevelopmental outcomes.

## Conclusions

Clinically inapparent significant episodes of IH are frequent in preterm infants in early postnatal age and they may persist to post-discharge. Knowledge of the association between IH and morbidity among all NICU caregivers would be of great benefit. Screening high-risk premature infants with continuous recordings of oxygen prior to discharge will help identify those who have severe episodes of IH to determine the appropriate treatment to be used.

## Appendices

Definitions

- Bronchopulmonary dysplasia (BPD; diagnosed according to the National Institute of Child Health and Human development criteria)

- Necrotizing enterocolitis (NEC) grade ≥ 2A; diagnosed and classified according to the modified Bell criteria

- Intraventricular hemorrhage (IVH) grade > 3

- Retinopathy of prematurity (ROP); diagnosed and graded according to the International Classification of Retinopathy of Prematurity

- Cystic periventricular leukomalacia (PVL); diagnosed when hypoechoic cysts were observed in the periventricular white matter)
